# Berberine Radiosensitizes Human Esophageal Cancer Cells by Downregulating Homologous Recombination Repair Protein RAD51

**DOI:** 10.1371/journal.pone.0023427

**Published:** 2011-08-08

**Authors:** Qiao Liu, Haiyan Jiang, Zhaojian Liu, Yu Wang, Minnan Zhao, Chunyan Hao, Shuai Feng, Haiyang Guo, Bing Xu, Qifeng Yang, Yaoqin Gong, Changshun Shao

**Affiliations:** 1 Key Laboratory of Experimental Teratology, Ministry of Education, and Institute of Molecular Medicine and Genetics, Shandong University School of Medicine, Jinan, Shandong, China; 2 Department of Pathology, Qilu Hospital of Shandong University, Jinan, Shandong, China; 3 Department of Breast Surgery, Qilu Hospital of Shandong University, Jinan, Shandong, China; The Chinese University of Hong Kong, Hong Kong

## Abstract

**Background:**

Esophageal squamous cell carcinomas (ESCC) have poor prognosis. While combined modality of chemotherapy and radiotherapy increases survival, most patients die within five years. Development of agents that confer cancer cell-specific chemo- and radiosensitivity may improve the therapy of ESCC. We here reported the discovery of berberine as a potent radiosensitizing agent on ESCC cells.

**Principal Findings:**

Berberine at low concentrations (<15 µM) substantially radiosensitized ESCC cells. X-ray induced DNA double-strand breaks (DSBs) persist longer in ESCC cells pretreated with berberine. Berberine pretreatment led to a significant downregulation of RAD51, a key player in homologous recombination repair, in ESCC cells, but not in non-malignant human cells. Downregulation of *RAD51* by RNA interference similarly radiosensitized the cancer cells, and, conversely, introduction of exogenous RAD51 was able to significantly counteract the radiosensitizing effect of berberine, thus establishing RAD51 as a key determinant in radiation sensitivity. We also observed that RAD51 was commonly overexpressed in human ESCC tissues, suggesting that it is necessary to downregulate RAD51 to achieve high radio- or chemotherapeutic efficacy of ESCC in clinic, because overexpression of RAD51 is known to confer radio- and chemoresistance.

**Conclusions/Significance:**

Berberine can effectively downregulate RAD51 in conferring radiosensitivity on esophageal cancer cells. Its clinical application as an adjuvant in chemotherapy and radiotherapy of esophageal cancers should be explored.

## Introduction

Esophageal squamous cell carcinomas (ESCC), which have a particularly high incidence in East Asian countries, usually have poor prognosis. Only a 4% 5-year survival rate was recorded with surgical care [1]. Radiotherapy had a similar outcome [2]. The survival rate of patients with ESCC can be significantly improved with combined chemotherapy and radiation therapy [3], [4]. However, increasing the radiation dose does not yield more satisfactory results [5]. There remains the necessity to develop agents that confer chemo- and radiosensitivity selectively on cancer cells while having minimal radiation toxicity to normal cells. Although various radiation sensitizers have been tested, including agents that target DNA or non-DNA targets, the ideal radiation sensitizer remains to be discovered [6].

Berberine, the main alkaloid component in Huang Lian and other medicinal herbs, is the most commonly used medicine for gastrointestinal discomfort in China. Beside its common use as a daily medicine, a number of laboratory studies have shown that berberine has antitumor activity for a wide variety of cancer cells, including glioblastoma [7], oral cancer [8], hepatoma [9], gastric cancer [10], prostate cancer [11], leukemia [12] and osteosarcoma [13]. In most cases, berberine was found to inhibit cell cycle progression and to induce apoptosis. A recent study showed that berberine was able to suppress the constitutive activation of NF-κB in some cancer cells [14], where it was found to directly inhibit IκB kinase (IKK) activation, leading to the suppression of phosphorylation and nuclear translocation of p65. Berberine was also reported to have a radiosensitizing effect on lung cancer cells by inducing autophagy [15]. In addition, berberine may impair tumor growth by inhibiting angiogenesis [16].

We previously showed that berberine may inhibit cancer cell proliferation by inducing DNA double-strand breaks (DSBs). Berberine, which emits a yellowish fluorescence, was found to be more intensively accumulated in the nuclei of osteosarcoma cells than in those of normal osteoblasts, and this differential accumulation of berberine correlates to its growth-inhibitory effect, to the induction of DNA damage, and subsequently to the activation of p53 and cell cycle arrest [13]. Therefore, the signal transduction pathway underlying the antitumor effect of berberine is probably similar to those initiated by other DNA-damaging agents such as cisplatin. A recent report showed that berberine can up-regulate p53 by disrupting the MDM2-DAXX-HAUSP interactions and promoting MDM2 self-ubiquitination and degradation [17].

We here report that berberine at low concentrations can significantly radiosensitize ESCC cells. We demonstrated that the radiosensitizing effect of berberine is mediated by the downregulation of *RAD51*, which encodes a critical protein in homologous recombination repair. In addition, we observed that *RAD51* was commonly over-expressed in human ESCC tissues, indicating that it is necessary to downregulate RAD51 to achieve high radio- or chemotherapeutic efficacy of ESCC in clinic, because overexpression of *RAD51* is known to confer radio- and chemoresistance.

## Results

### Berberine sensitizes esophageal cancer cells to ionizing radiation

**Figure 1:**
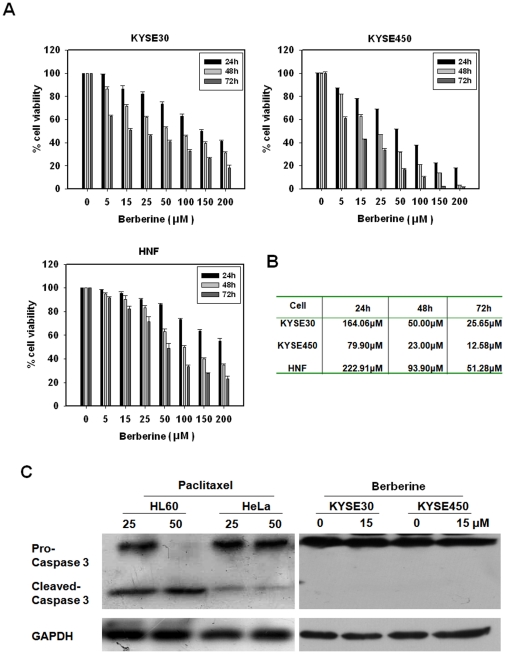
Growth-inhibitory effect of berberine on esophageal cancer cells. (A), Effect of berberine on the viability of KYSE30, KYSE450 and HNF cells. Cell viability was assessed by MTT assay. Cells were treated with DMSO or the indicated concentrations of berberine for 24 h, 48 h and 72 h. Results were represented as mean ± SD from three independent experiments. (B), Cytotoxicity of berberine against KYSE30, KYSE450 and HNF cells represented by inhibitory concentrations IC50. (C), Measurement of apoptosis by Western blot analysis of caspase-3. KYSE30 and KYSE450 cells were treated with berberine for 24 h and 48 h, respectively. Paclitaxel-treated HL-60 and HeLa cells as positive control.

**Figure 2:**
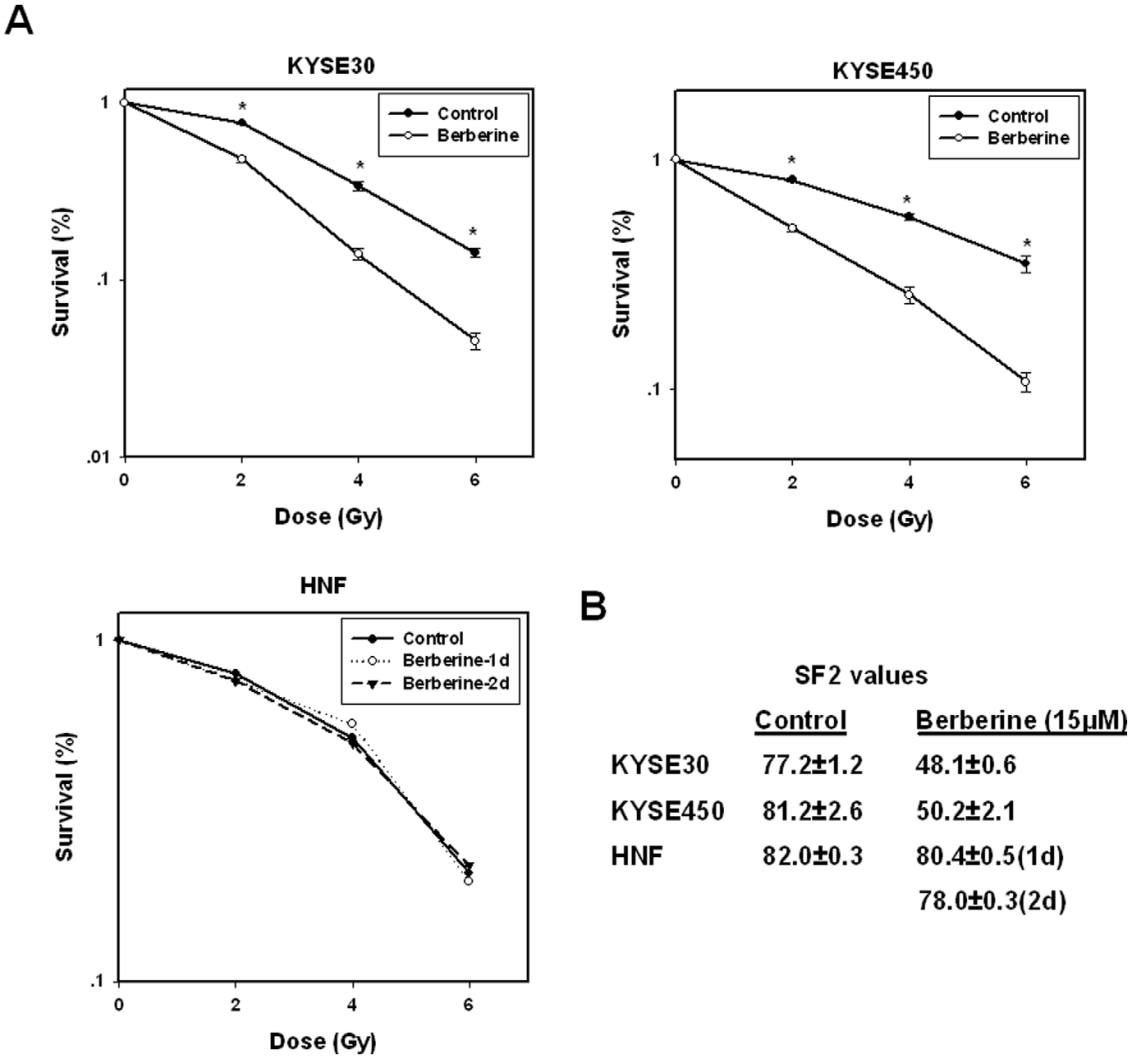
Radiosensitization of esophageal cancer cells by berberine. (A), Radiosensitization by berberine by clonogenic cell survival assay. KYSE30 and KYSE450 cells were exposed to berberine (15 µM) or vehicle control (DMSO) for 24 and 48 h, respectively, before they were irradiated and plated. Human normal fibroblast cells were treated with berberine for 24 h and 48 h before they were exposed to X-rays. Data points are the averages of two independent experiments each plated in triplicate; bars, SE. (B), SF2 values following treatment with berberine. Decrease in the SF2 values indicates the radiosensitizing effects of berberine.

As previously reported for other types of cancer cells, berberine treatment alone had a growth-inhibitory effect on esophageal cancer cells in a dose- and time-dependent manner (Fig. 1A). When compared to human normal fibroblasts (HNF), ESCC cells are more sensitive to berberine. At 72 h, the IC50 for ESCC cells KYSE450 (12.58 µM), was about a quarter of that for HNF (51.28 µM) (Fig. 1B). At low concentrations (<15 µM), berberine had a relatively low toxicity to ESCC cells and induced no detectable apoptosis (Fig. 1C). However, ESCC cells that were pretreated with low concentrations of berberine exhibited a significantly increased sensitivity to ionizing radiation, as reflected by the remarkable decrease in their ability to form colonies (Fig. 2). Berberine pretreatment (15 µM, 24 h) reduced the SF2 (surviving fraction at 2 Gy) of KYSE30 cells to 48.1±0.6%, from 77.2±1.2% in cells that were not pretreated with berberine. A second ESCC line KYSE450 showed a similar response, with the SF2 reduced to 50.2±2.1% when cells were pretreated with berberine at 15 µM for 48 h, compared to 81.2±2.6% for cells not pretreated with berberine. In contrast, berberine had no radiosensitizing effect on human normal fibroblasts (Fig. 2).

### Berberine pretreatment prolongs the persistence of radiation-induced DSBs

**Figure 3:**
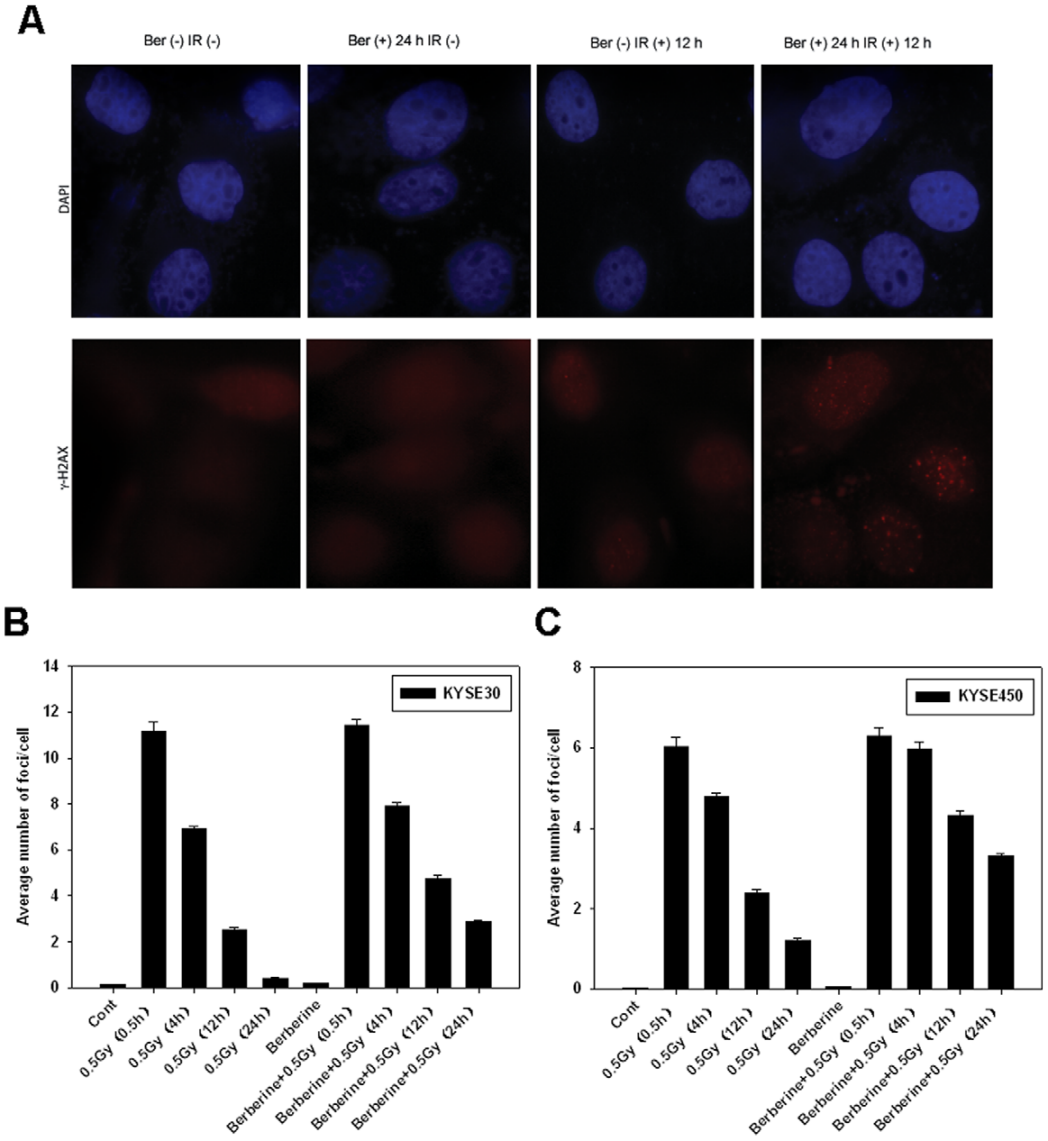
Berberine pre-treatment led to a prolonged persistence of IR-induced γ-H2AX foci. (A), Immunofluorescence staining of γ-H2AX in KYSE30 cells. (B), Summary of γ-H2AX foci in KYSE30 cells at different time points following various treatments. (C), Summary of γ-H2AX foci in KYSE450 cells at different time points following various treatments. Columns, means of three independent experiments. Cells growing on coverslips in 6-well plates were exposed to berberine (15 µM) for 24, irradiated (0.5 Gy), and fixed at the specified time points for immunofluorescence analysis.

Ionizing radiation kills cells by inflicting various types of damage to the genome. We speculated that the radiosensitizing effect of berberine on cancer cells may be caused by impairment in the repair of DNA double-strand breaks (DSBs). We therefore determined the levels of DSBs by immunofluoresence staining of γ-H2AX foci in ESCCs at different time points after exposure to X-rays. As shown in Fig. 3A, in KYSE30 cells not pretreated with berberine, majority of the γ-H2AX foci were cleared at 12 h after exposure to 0.5 Gy of X-rays, and the average number of γ-H2AX foci subsided to near basal level at 24 h. In contrast, more γ-H2AX foci persisted at those two time points in KYSE30 cells that were pretreated with berberine (15 µM). The average number of γ-H2AX foci per cell in cells receiving the combined berberine/radiation treatment was significantly greater compared with the radiation-only group at 12 and 24 h time points (Fig. 3B) (*P* = 0.002, and *P*<0.001, respectively). Berberine treatment alone was not observed to induce obvious DNA damage in terms of γ-H2AX foci induction over untreated controls (*P* = 0.081).

KYSE450 cells also showed a delayed clearance of the X-ray-induced γ-H2AX foci when they were pretreated with berberine. While the majority of γ-H2AX foci were cleared in control cells at 24 h after exposure to X-rays, more than half of the γ-H2AX foci persisted in cells that were pretreated with berberine (Fig. 3C). These results indicate that berberine pretreatment impaired the repair of x-ray-induced DSBs.

### Berberine downregulates RAD51 in cancer cells but not in nonmalignant cells

**Figure 4:**
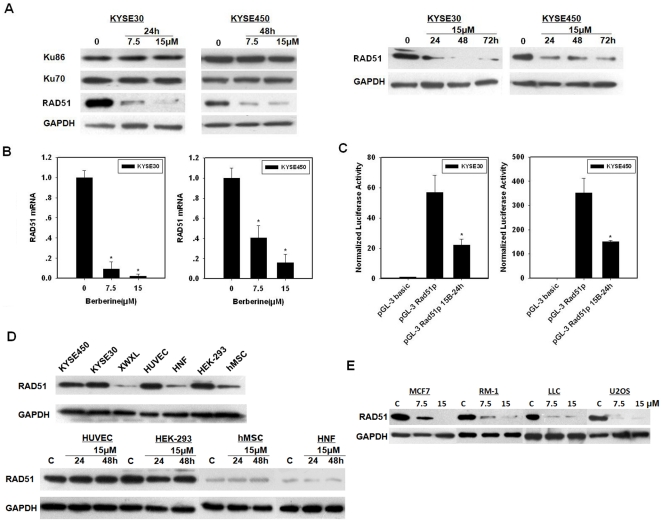
Berberine downregulates RAD51 in tumor cells. (A), The levels of DNA repair proteins in esophageal cancer cells treated with berberine. Cells were treated with berberine at the indicated concentrations and for the indicated duration before cells were prepared for cell extracts. (B), Decrease in *RAD51* mRNA levels after berberine exposure. Cells were exposed to berberine (15 µM) for 24 h for KYSE30 cells (48 h for KYSE450 cells), and collected for quantitative real-time PCR analysis of the RAD51/GAPDH mRNA ratios. (C), *RAD51* promoter activity was suppressed by berberine in KYSE30 and KYSE450 cells. The pGL3-RAD51p luciferase (firefly) reporter plasmids were transiently transfected into human esophageal cancer cells. Four h later, the cells were treated with berberine for 24 h, and then assayed for luciferase activity. Firefly luciferase values were normalized to Renilla luciferase activity from a co-transfected pRL-SV40 control vector. Each value represents the mean SE for three independent experiments. Statistical analysis was performed using unpaired Student's t test. *, P<0.05 as compared with control values. (D), Lack of RAD51 downregulation in nonmalignant cells. Cells were exposed to berberine (15 µM) for the specified time period and collected for immunoblot analysis. (E), Downregulation of RAD51 in other types of cancer cells. Cells were exposed to the designated berberine concentration for 48 h and collected for immunoblot analysis. The immunoblots shown in A, D, and E are representative of at least two independent experiments with GAPDH serving as a protein loading control. C indicates cultures exposed to the vehicle control DMSO.

DSBs are primarily repaired by two pathways, i.e., non-homologous end joining and homologous recombination [18], [19], [20]. Ku70 and Ku86 are essential for the former, where as RAD51 is a central player in the latter. We next determined whether the levels of those three proteins were changed in esophageal cancer cells pretreated with berberine. We observed that while there were no obvious changes in the levels of Ku70 and Ku86, the level of RAD51 protein was remarkably decreased in ESCCs that were treated with berberine for 24 h or 48 h at 7.5 µM or 15 µM (Fig. 4A). The level of *RAD51* mRNA showed a significant reduction in both cancer cell lines after treatment with berberine (Fig. 4B), suggesting that the downregulation of RAD51 was probably due to decreased transcription of *RAD51* mRNA.

The expression of RAD51 is known to be cell cycle-dependent and is higher at S and G2/M phases than at G0/G1 phase [21], [22]. To determine whether the reduced expression of RAD51 was due to change in cell cycle distribution in berberine-treated cells, we subjected berberine-treated cells to flow cytometry analysis. While berberine treatment (15 µM for 24 h) reduced the percentage of KYSE30 cells at S and G2/M to 49% from 60% in control (data not shown), such a change was not enough to account for the many fold reduction in the level of RAD51 expression. More importantly, berberine treatment (15 µM for 24 h) did not significantly alter the distribution of cell cycle distribution of KYSE450 cells (44% and 45% for the percentage of cells at S-G2/M phases in control and treated group, respectively), indicating that the reduction of RAD51 expression in response to berberine is not due to reduced population size of cells at S and G2/M phases. Thus, the downregulation of RAD51 in response to berberine is probably independent of the expression changes that are associated with cell cycle progression.

Overexpression of *RAD51* is frequently associated with an increase in its promoter activity [23]. We next cloned the promoter and the 5′end region of *RAD51* (from −1693 to +487 bp) into a luciferase reporter plasmid, pGL3-basic vector, transfected them into esophageal cancer cells, and tested the *RAD51* promoter activity in response to berberine treatment. As shown in Fig. 4C, the promoter activity of *RAD51* was significantly reduced in cells that were treated with berberine when compared to control. Together, these results showed that berberine was able to downregulate the expression of *RAD5*1 in esophageal cancer cells at transcription level.

Because RAD51 is a key player in homologous recombination repair, its downregulation could contribute to the radiosensitivity of ESCC cells pretreated with berberine. Since berberine had no radiosensitizing effect on HNF, we speculated that the RAD51 level might not be affected by berberine treatment in HNF. We first surveyed the basal levels of RAD51 in several nonmalignant cell lines including HNF. As shown in Fig. 4D, the levels of RAD51 were much lower in HNF, hMSC (human mesenchymal stem cells derived from umbilical cord) and XWXL (fibroblasts derived from a benign fibroadenoma) than those in ESCC cells. Interestingly, human umbilical vascular endothelial cells (HUVECs) and HEK-293 cells also expressed high levels of RAD51. However, in contrast to ESCC cells, none of the four types of nonmalignant cells showed a downregulation in RAD51 expression upon berberine treatment (Fig. 4D).

To determine whether the downregulation of RAD51 by berberine applies to other types of cancer cells, we also exposed human breast cancer cells MCF7, human osteosarcoma cells U2OS, mouse prostate cancer cells RM-1 and mouse lung cancer cells LLC to low concentrations of berberine for 48 h and measured the protein level of RAD51. As shown in Fig. 4E, RAD51 was downregulated in all those four cancer cell lines tested, suggesting that downregulation of RAD51 by berberine is not unique to ESCC cells. Together, these results indicate that different types of cells may respond differently to berberine treatment in terms of regulation of RAD51 expression.

### Upregulation of RAD51 by IR can be attenuated by berberine

**Figure 5:**
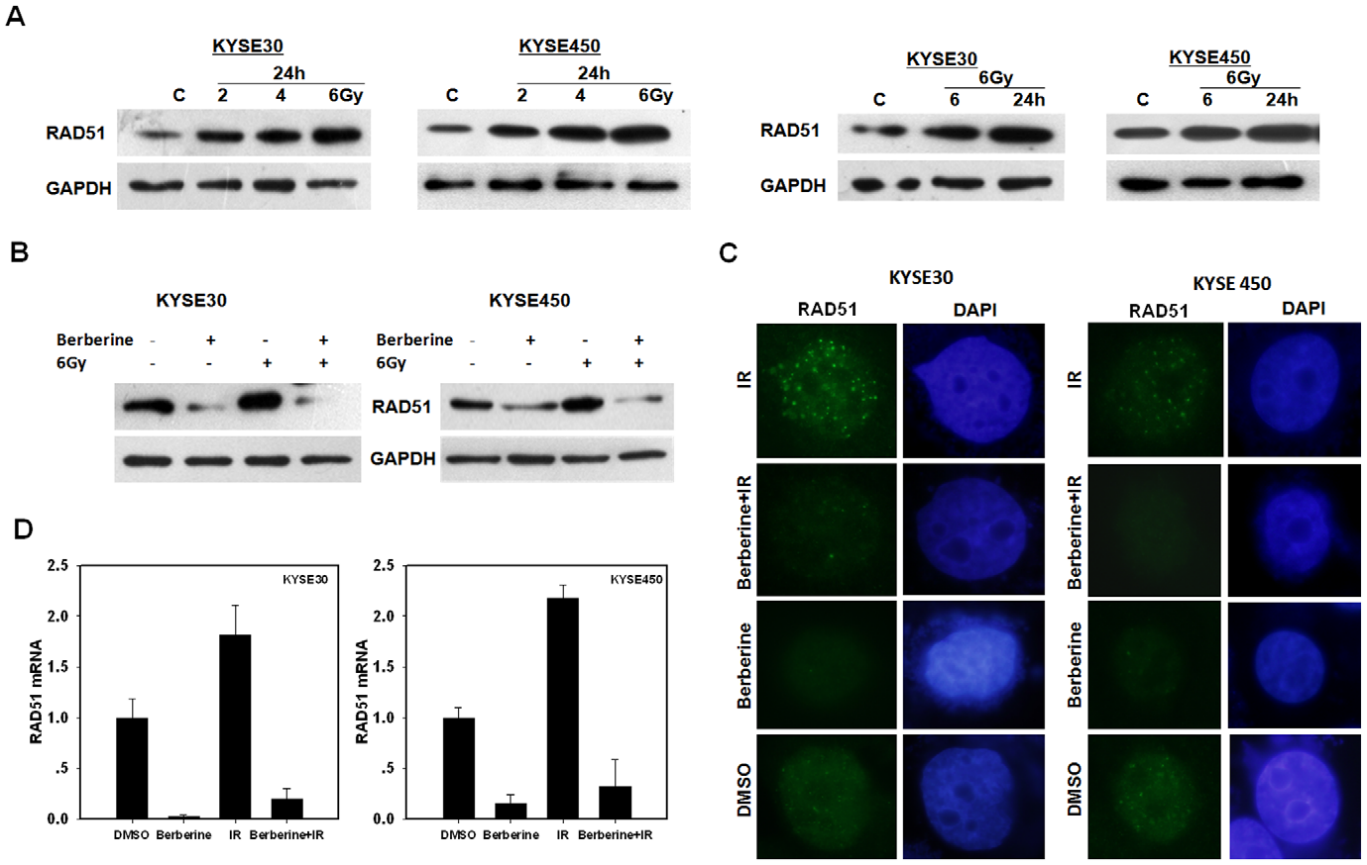
Upregulation of RAD51 by IR can be attenuated by berberine. (A), Radiation enhances RAD51 expression in tumor cells. Cells were exposed to the indicated doses of radiation and collected 24 h later for immunoblot analysis. (B), Change of RAD51 protein levels in response to berberine and radiation. Cells were pretreated with berberine (15 µM) for 24 h before being irradiated with 6 Gy of X-rays. Cells were collected for immunoblot analysis at 24 h after irradiation. (C), Inhibition of radiation-induced RAD51 foci formation by berberine. Shown are immunofluorescence images of KYSE30 and KYSE450 cells stained with anti-RAD51 antibody after 24 h of pretreatment with berberine and at 24 h after irradiation with 6 Gy of X-rays. DAPI counterstaining showed the nuclei. (D), *RAD51* mRNA levels after pretreatment with berberine (15 µM) for 24 h (KYSE30) or 48 h (KYSE450) before irradiation with 6 Gy. Cells were collected for quantitative real-time PCR analysis of the RAD51/GAPDH mRNA ratios at 24 h after exposure to 6 Gy of X-rays. Immunoblots and mRNA expression values are representative of at least two independent experiments.

We observed that ionizing radiation induced an up-regulation of RAD51 in ESCC cells (Fig. 5A). In consistent with the increase of RAD51 at protein level, the level of *RAD51* mRNA was increased in ESCC cells that had been exposed to IR (data not shown). This finding is in accordance with a previous report of RAD51 induction by IR in glioma cells [24]. Because IR can upregulate the expression of *RAD51*, we next tested whether berberine pretreatment could counteract this effect of IR. The ESCC cells were first treated with berberine, then with IR and the level of RAD51 expression was measured. In contrast to the elevation of RAD51 in cells that were treated with IR alone, berberine pretreatment effectively attenuated the up-regulation of RAD51 induced by IR (Fig. 5B), indicating that berberine was able to overcome the upregulation of RAD51 induced by IR. Not only was there a reduction in total protein level, the formation of RAD51 foci in response to IR was greatly inhibited by berberine (Fig. 5C). The downregulation of RAD51 by berberine was again reflected at transcription level (Fig. 5D).

### RAD51 downregulation by RNAi radiosensitizes cancer cells

**Figure 6:**
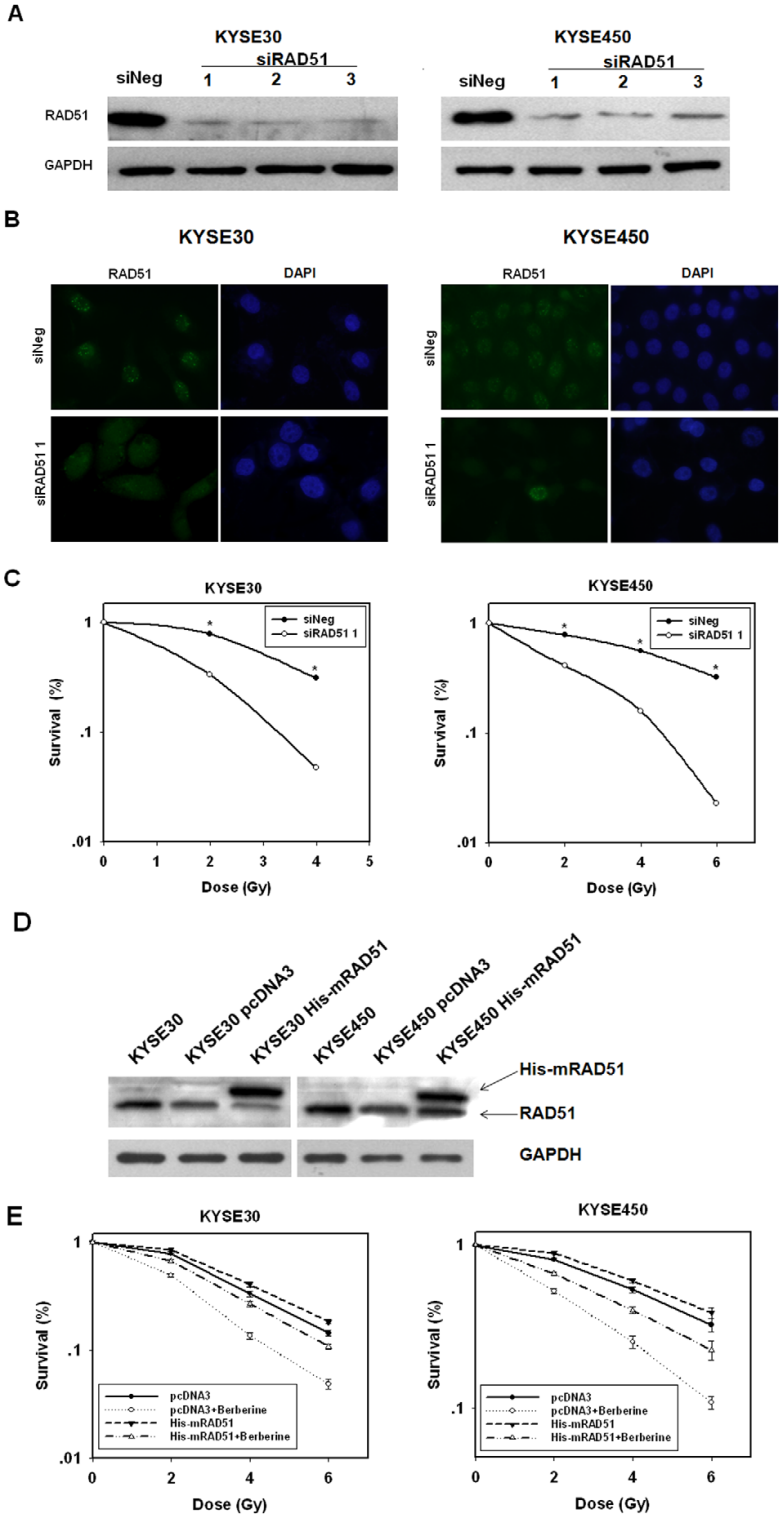
RAD51 expression level determines radiosensitivity of ESCC cells. (A), Downregulation of RAD51 by siRNA. RAD51 protein levels were measured by Western blot. KYSE30 and KYSE450 cells were transfected with siRNA duplexes (200 nM) specific to *RAD51* or negative oligo in serum-free medium for 4 h, then replaced with complete medium for 24 h. Whole cell extracts were collected for western blot analysis using RAD51 antibodies. (B). Reduced formation of radiation-induced RAD51 foci in esophageal cancer cells in which *RAD51* was silenced by RNAi. Cells were exposed to 6 Gy of X-rays at 28 h following transfection with siRNA duplexes. (C), Effect of RAD51 knockdown on radiosensitivity of esophageal cancer cells as determined by clonogenic survival assay. (D), Western blot analysis of RAD51 protein in cancer cells that were transfected with pcDNA3.1 or pcDNA3.1B-mRAD51. The immunoblots shown are representative of two independent experiments with GAPDH serving as a protein loading control (KYSE30/KYSE450 parent cell line, KYSE30/KYSE450 (pcDNA3.1) cells stably transfected with pcDNA3.1, KYSE30/KYSE450 (mRAD51) cells stably transfected with pcDNA3.1B-mRAD51). (E), Effect of RAD51 overexpression on radiosensitivity of esophageal cancer cells as determined by clonogenic survival assay. The results for the cells transfected with the mRAD51 or pcDNA3.1 vector treated with berberine were compared (*P<0.01).

Because berberine may affect many cellular pathways that are involved in cell proliferation and survival, it cannot be ruled out that pathways other than the downregulation of RAD51 were responsible for the radiosensitizing effect of berbeirne. To demonstrate that it is the downregulation of RAD51 that is responsible for this effect of berberine, we next depleted RAD51 in esophageal cancer cells by RNAi and tested their radiosensitivity. As shown in Fig. 6A, *RAD51* expression could be efficiently silenced by siRNA specifically targeting *RAD51* in both cell lines. In addition, the spontaneous formation of RAD51 foci was greatly reduced (Fig. 6B). As expected, the depletion of RAD51 led to a significant reduction in the clonogenic cell survival for both esophageal cancer cell lines (Fig. 6C). The SF2 was reduced to 33.3±0.4% in RAD51-RNAi KYSE30 cells from 78.4±1.2% in control. KYSE450 cells showed a similar response to RAD51 depletion. Thus, the radiosensitizing effect of berberine on esophageal cells was primarily mediated by the downregulation of RAD51.

### Introduction of Exogenous RAD51 counteracts the radiosensitizing effect of berberine

To further validate the role of RAD51 as a key player in mediating the radiosensitizing effect of berberine, we made a construct that expresses murine RAD51 under the CMV promoter, pcDNA3.1B-mRAD51, and had ESCC cells stably transfected. Expression of the exogenous RAD51 was evident in cells transfected with mRAD51, as shown by Western blot (Fig. 6D). Importantly, the radiosensitizing effect of berberine was significantly attenuated in both KYSE30 and KYSE450 cell lines that were transfected with mRAD51 (Fig. 6E). These results, together with those obtained with RAD51-RNAi, demonstrated that RAD51 is a critical determinant of radioresistance in ESCC cells.

### RAD51 overexpression is common in esophageal cancer tissues

**Figure 7:**
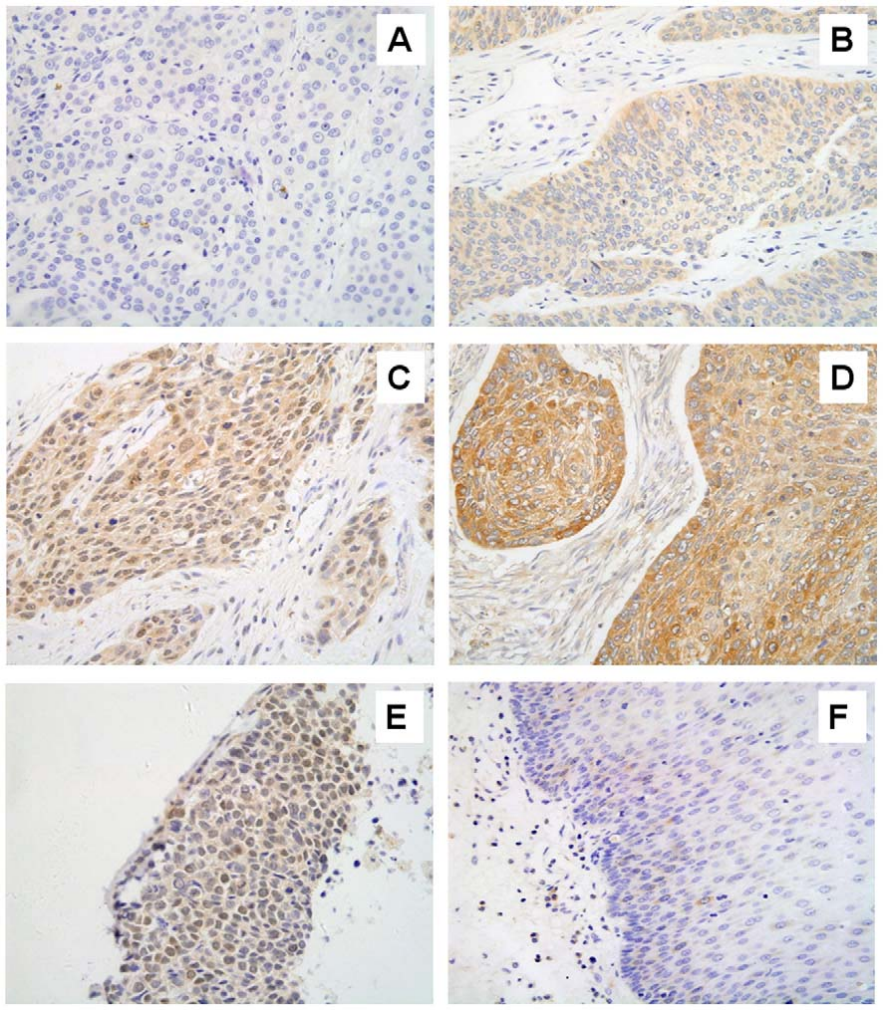
RAD51 is up-regulated in human esophageal cancer specimens. Images of showing different expression level and cellular distribution are shown. (A), absence of RAD51-specific staining (blue nuclei due to Mayer's Hemalum counterstaining). (B), low levels of RAD51 in the cytoplasm, but not in nuclei, of the invasive carcinoma cells. (C), intermediate levels of RAD51 in both the cytoplasm and nuclei of the invasive carcinoma cells. (D), high levels of RAD51 in the cytoplasm, but not in nuclei, of the invasive carcinoma cells. (E), high levels of nuclear RAD51 in the nuclei of invasive carcinoma cells. No cytoplasm staining is evident. (F), absence of RAD51 staining in normal esophageal tissue. Immunohistochemistry was done as in Materials and Methods. All original images were captured at×400 magnification.

Upregulation of *RAD51* expression is common in tumor cell lines [25] and in a number of human malignancies [26], [27], [28]. We next evaluated the *RAD51* expression level in a panel of 86 human primary esophageal squamous cell carcinoma tissues by immunohistochemical staining. Nuclear and/or cytoplasmic staining of RAD51 was detected in 81 of 86 specimens. In specimens exhibiting positive staining of RAD51, subcellular distribution and intensity were varied. Intensity was scored as 1 in 29, 2 in 43, and 3 in 9. Six samples showed predominant nuclear staining, 30 samples showed predominant cytoplasmic staining, and 45 samples showed both cytoplasmic and nuclear staining (Fig. 7A–E). In contrast, *RAD51* expression was low or absent in normal tissues surrounding the carcinoma (Fig.7F). However, we found no correlation between RAD51 expression levels and the clinicopathological parameters in ESCCs (Table 1). Nevertheless, our results showed that RAD51 is commonly overexpressed in ESCC specimens. Because overexpression of RAD51 usually confers resistance to agents that damage DNA [29], [30], [31], our results call for measures that downregulate RAD51 in order to attain high radio- or chemotherapeutic efficacy of ESCCs in clinical settings. Interestingly, berberine treatment also down-regulated RAD51 in three additional esophageal cancer cell lines (KYSE410, EC109, and TE-1), as in KYSE30 and KYSE450 cells (data not shown), suggesting that the berberine may render radiosensitizing effect to a broad array of esophageal cancer cells.

**Table 1:** Lack of association between RAD51 levels and the clinicopathological parameters in ESCC specimens.

Characteristic	Category	N = 86 (%)	RAD510 1 2 3	*P*-value
GenderAge (years)Tumor differentiationDepth of invasionTumor size (cm)Lymph node statusStage	MaleFemale≥60<60WellModeratePoorT1-T2T3-T4≥5<5negativepositiveLow (I and II)High (III and IV)	73(81.4%)13(18.6%)45(52.3%)41(47.7%)16(18.6%)39(45.3%)30(36.1%)23(26.7%)63(73.3%)23(26.7%)63(73.3%)39(45.4%)47(54.6%)10(11.6%)76(88.4%)	4 26 35 81 3 8 14 18 18 51 11 25 41 5 9 12 9 24 42 15 10 41 9 12 14 20 31 82 9 10 23 20 33 72 12 22 33 17 21 60 6 4 05 23 39 9	0.4060.1990.4460.3060.3060.3060.306

## Discussion

We demonstrated for the first time that berberine can efficiently downregulate RAD51 expression in cancer cells in conferring radiosensitivity. Berberine treatment led to a reduction in *RAD51* transcription and an inhibition of *RAD51* promoter activity. It can also overcome the upregulation of RAD51 that is induced by ionizing radiation (IR). The radiosensitizing effect of berberine was attenuated in cancer cells overexpressing exogenous RAD51. Downregulation of RAD51, which is essential for homologous recombination repair, greatly impaired the repair of DSBs induced by IR and led to poor survival of IR-treated cells. Importantly, we observed that RAD51 was commonly upregulated in human ESCC tissues, which may render resistance to therapies that target DNA. Therefore, administration of berberine as a radio- and chemosensitizer may serve as a strategy in the treatment of ESCC patients.

Upregulation of RAD51 in cancer cells was shown to be associated with increased chemoresistance [29], [30]. Several previous studies also showed that some anti-cancer drugs render cancer cells radiosensitive and chemosensitive by downregulating RAD51 and consequently impairing homologous recombination repair in cancer cells. For example, imatinib (Gleevec), which inhibits c-Abl tyrosine kinase, can efficiently reduce the expression of RAD51 in cancer cells and confer radiosensitivity [24], [32]. Gefitinib, a selective epidermal growth factor receptor tyrosine kinase inhibitor, was found to downregulate RAD51 in lung cancer cells and sensitize them to mitomycin C [33] and gemcitabine [34]. Phenyl hydroxamic acid PCI-24781, a histone deacetylase inhibitor that has a radiosensitizing effect on cancer cells, also acts by downregulating RAD51 [35]. Besides having an array of side effects, these drugs tend to be very costly.

Many natural products have remarkable medicinal values. Besides being more affordable, herbal medicines usually have a low toxicity. Several natural compounds have been found to confer radiosensitivity and chemosensitivity on cancer cells. One study showed that curcumin, a component in turmeric (*Curcuma longa*), could radiosensitize prostate cancer cells via the inhibition of NFκB function, which led to a downregulation of the anti-apoptotic gene *Bcl-2* [36]. Treatment of cervical cancer cells with curcumin led to enhanced production of IR-induced reactive oxygen species (ROS), which causes sustained ERK1/2-MAPK activation [37]. In contrast, emodin, a substance found in some plants including rhubarb, was reported to inhibit the activation of ERK1/2 in lung cancer cells, which in turn leads to a downregulation of RAD51 and confers chemosensitivity [38], [39].

Huang Lian is one of the most commonly used medicinal herbs in China. It is one of the 12 most commonly used medicinal herbs in the United States [40]. Besides its common use as a drug for gastrointestinal discomfort, berberine has been tested in clinical trials for type 2 diabetes mellitus [41], [42] and on hypercholesterolemia [43]. Importantly, patients taking berberine experience relatively mild side effects. While previous studies showed that berberine possesses direct anti-tumor effects by inducing the production of reactive oxygen species in cancer cells, which would presumably confer radiosensitization [44], [45], [46], [47], our study is the first to show that berberine can confer radiosensitivity by downregulating a key player in the repair of DSBs. It should be noted that the potency of berberine in downregulating RAD51 in ESCC cells is comparable to that of Gleevec in glioma cells [24]. At higher concentrations, berberine is expected to play a dual role in promoting the death of the cells in which it is retained, by simultaneously inflicting DNA damage and impairing a pathway that is responsible for the repair of the damage. Importantly, at concentrations that confer radiosensitivity to esophageal cancer cells, berberine had no radiosensitizing effect on normal human fibroblasts. Correspondingly, berberine was not observed to downregulate RAD51 in nonmalignant cells. These results suggest that the radiosensitizing effect of berberine may be specific to cancer cells.

In summary, we showed that berberine at low concentrations substantially radiosensitized esophageal squamous cell carcinoma cells. It acts by downregulating RAD51, a key player in homologous recombination repair, leading to impairment in the repair of double-strand breaks. Our findings thus uncovered a hitherto unrecognized biological activity of a commonly used and inexpensive drug and indicated that berberine, with its relatively low toxicity, may be used as an adjuvant in cancer radiation therapy.

## Materials and Methods

### Ethics Statement

All procedures involving clinical samples were approved by the Ethics Committee of Shandong University School of Medicine.

### Cell Lines

All cells were grown at 37°C in humidified incubator containing 5% CO_2_. The human esophageal squamous cell carcinoma (ESCC) lines KYSE30, KYSE450, KYSE410, EC109 and TE-1 were obtained from Cancer Institute & Hospital, Chinese Academy of Medical Sciences (Beijing) and cultured in RPMI 1640 supplemented with 10% fetal bovine serum, 100 units/mL penicillin, and 100 µg/mL streptomycin. KYSE30, KYSE450 and KYSE410 were originally generated by Dr. Yutaka Shimada, Normal human fibroblast (NHF) cells were obtained from the Institute of Basic Medical Sciences, Chinese Academy of Medical Sciences (Beijing), and were maintained in DMEM supplemented with 10% fetal bovine serum, 100 units/mL penicillin-streptomycin and 4 µg/mL bFGF. HUVECs were obtained from ATCC. Mesenchymal stem cells derived from human umbilical cord were a gift from Dr. Dong Li, Qilu Hospital, Shandong University. Fibroblasts (XWXL) were established from a surgically removed fibroadenoma, with informed consent of the patient and approved by Ethics Committee of Shandong University School of Medicine. The other cell lines used in this study were obtained from the Cell Bank of Chinese Academy of Sciences (Shanghai).

### Chemicals

The chloro-derivative of berberine was from Sigma Chemical (St Louis, MO). We refer to berberine chloride as berberine hereinafter. A 50 mM stock solution was prepared in DMSO and stored at −80°C in aliquots until use. MTT, (3-[4,5-dimethyl-2-yl]-2,5-diphenyl tetrazolium bromide), and all other chemicals were of analytical grade and purchased from Sigma Chemical.

### MTT assay

Cells in early log phase were trypsinized and plated in 96-well cell culture plates at the concentration of 2−5×10^3^ cells/well. Twenty-four h later, the medium was removed and replaced with fresh medium with or without berberine. Cell density was measured on day 1, 2 and 3 by using the MTT following the manufacturer's instructions. The absorbance of converted dye is measured at the wavelength of 490 nm and the absorbance is directly proportional to cell viability. For these studies, all experiments were repeated three or more times.

### Clonogenic survival assay

The survival and proliferation potential of cells treated with berberine and/or ionizing radiation were assessed by clonogenic assays. Briefly, the cells were treated with the vehicle control (DMSO) or 15 µM berberine for the indicated time and then irradiated using a Faxitron Cabinet X-ray System (Faxitron X-ray Corp., Wheeling, IL) to deliver the indicated doses at room temperature. The X-rays were filtered through a 0.5 mm aluminum filter resulting in a dose rate of 0.4 Gy/min. Cells were trypsinized, suspended in complete medium, counted and replated in 100-mm tissue culture dishes to allow formation of macroscopic colonies. Plates were incubated at 37°C for 7 to 14 days, fixed with methanol, stained with Giemsa, and colonies containing at least 50 cells in size were counted. The fraction surviving a given x-ray dose was calculated based on the survival of nonirradiated cells treated with the vehicle or berberine.

### Immunofluorescence staining of γ-H2AX and Rad51

Cells were grown on coverslips in 6 well plates and treated with berberine and/or X-ray. At specified times, medium was aspirated, washed in PBS thrice and cells were fixed in 4% paraformaldehyde for 15 minutes at room temperature, followed by treatment with 0.2% Triton X-100 in PBS for 5 minutes. Cells were then washed in PBS twice and then blocked with 5% bovine serum albumin in PBS for 30 minutes, following which mouse anti-γ-H2AX antibody (Millipore, Billerica, MA) or rabbit anti-Rad51 antibody (Santa Cruz, CA) was added at a dilution of 1∶600 and 1∶300 in 5% bovine serum albumin in PBS respectively and incubated overnight at 4°C. Cells were then washed thrice in PBS before incubating in the dark with a Rhodamine-labeled or FITC-labeled secondary antibody at a dilution of 1∶300 in 5% bovine serum albumin in PBS for 60 minutes. The secondary antibody solution was then aspirated and the cells were washed four times in PBS. Cells then were incubated in the dark with 4′,6-diamidino-2-phenylindole (1 µg/mL) in PBS for 5 minutes and coverslips were mounted with an antifade solution (Molecular Probes, Eugene, OR). Slides were then examined on a Leica fluorescent microscope. Images were captured by a charge coupled device camera. For each treatment condition, γ-H2AX foci were counted in at least 100 cells from randomly captured images.

### Western Blot Analysis

Cells were harvested after treatment with berberine and/or X-ray, rinsed in ice-cold PBS, and lysed in lysis buffer containing 50 mmol/L HEPES (pH 7.9), 0.4 mol/L NaCl, 1 mmol/L EDTA, 2 µg/mL leupeptin, 2 µg/mL aprotinin, 5 µg/mL benzamidine, 0.5 mmol/L phenylmethylsulfonylfluoride, and 1% NP40. The lysates were centrifuged at 12,000 rpm to remove any cellular debris. Protein concentrations of the lysates were determined by the BCA protein assay system (Beyotime, China). Equal amounts of protein were separated by 10% SDS-PAGE, transferred to PVDF membrane (Millipore, Billerica, MA), and blocked with 5% nonfat dry milk in TBS-Tween 20 (0.1%, v/v) for 1 hour at room temperature. The membrane was incubated with primary antibody overnight. Antibodies to Ku70, Ku86, and RAD51 were from Santa Cruz Biotechnology (Santa Cruz, CA); and GAPDH was from Chemicon (Temecula, CA). After washing, the membrane was incubated with the appropriate horseradish peroxidase secondary antibody (diluted 1∶5,000; Amersham Pharmacia Biotech, Arlington Heights, IL) for 1 hour. Following several washes, the blots were developed by enhanced chemiluminescence (Millipore, Billerica, MA).

### cDNA synthesis and real-time PCR

RNA was isolated using TRIzol reagent (Invitrogen) according to the manufacturer's protocol. cDNA was synthesized by reverse transcription of 1 µg of total RNA with random hexamers. The total volume of reverse transcription reaction was 20 µl. Real-time quantitative reverse transcription-PCR was performed using the ABI Prism 7500 sequence Detection System (Applied Biosystems, Inc., Foster City, CA). Human GAPDH gene was used as an internal control. The levels of *RAD51* and *GAPDH* mRNA were measured by SYBR Green I assay. *RAD51* was amplified by using the primers with the sequence 5′-CAACCCATTTCACGGTTAGAGC-3′ (forward) and 5′-GCTTTGGCTTCACTAATTCCCTT-3′(reverse) in conjunction with thermal cycling program consisting of one cycle for 2 min at 50°C and for 10 min at 95°C, and 40 cycles for 15 s at 95°C and for 1 min at 60°C. 26 cycles of 95°C for 30 s, 61°C for 30 s and 72°C for 60 s. Glyceraldehyde 3-phosphate dehydrogenase (GAPDH) was amplified as an internal control. The GAPDH primer was 5′- CAGAACATCATCCCTGCCTCTAC-3′ (forward) and 5′- TTGAAGTCAGAGGAGACCACCTG-3′ (reverse). The samples were loaded in quadruple, and the results of each sample were normalized to GAPDH.

### Luciferase reporter assay

The sequence of promoter and 5′end of *RAD51* (from −1693 to +487 bp of the transcriptions start site-numbered according to Entrez Gene ID 5888, gi:19924132) was cloned into pGL3-basic vector. Primers are available upon request. For transfection, 5×10^5^ KYSE30 and KYSE450 cells were seeded in duplicate into 6-well culture plates and transfected with 1 µg of reporter construct using lipofectamine 2000 (Invitrogen) per the manufacturer's instructions. Cells were incubated with/without berberine (15 µM) for 24 h then harvested. Firefly and Renilla luciferase activities were measured by using the Dual-Luciferase Reporter Assay System kit (Promega) and assessed with a luminometer. Renilla luciferase activity from a cotransfected pRL-SV40 control vector (50 ng/well) was used for normalization.

### RAD51 depletion by RNA interference

*RAD51* siRNA duplex 1, 2 and 3 and the negative oligo were obtained from GenePharma (Shanghai, China). siRNA duplexes or negative oligo (200 nM) were transfected into KYSE30 and KYSE450 cells using lipofectamine2000 (Invitrogen) for 24 h. RAD51 protein levels were determined by western blotting analysis and the levels of radiosensitivity were determined by clonogenic assay described above.

Sequences for small interfering RNA for *RAD51* RNAi are as follows:

siRAD51-1: CGAUGUGAAGAAAUUGGAATT

siRAD51-2: GACUGGAUCUAUCACAGAATT

siRAD51-3: GCAGUGAUGUCCUGGAUAATT

### Expression vector of mRAD51

Plasmid pcDNA3.1B-mRAD51 was constructed by PCR-amplifying the cDNA of mRAD51 from the pCMV-SPORT6- mRAD51 cDNA plasmid (Open Biosystems, Huntsville, AL) with primers: GCGGGATCCACTATGGCTATGCAAATGCAGCTTGAAG and TATCTCGAGCGGTCTTTGGCATCGCCCACTCCATCTG. After a double cut with *Bam*H I and *Xho* I, the PCR product was inserted into a pcDNA3.1/*myc*-His B vector (Invitrogen, Carlsbad, CA, USA) that was opened by the same restriction enzymes. The mRAD51 insert was verified by sequencing.

### Immunohistochemistry

ESCC specimens were from the collection in the Department of Pathology, Qilu Hospital. The use of the specimens was approved the Ethics Committee of Shandong University School of Medicine. Immunohistochemical staining was performed using the PV6000 IHC Kit (Zhongshan Goldenbridge Biotechnology Co. Ltd., China). Briefly, paraffin sections (4 µm) of human ESCC samples were heated, dewaxed, and hydrated in xylene and ethanol/H_2_O. Antigen was retrieved by microwave oven heating (10 min) at middle power in 0.01 mol/L sodium citrate buffer (pH 6.0). The sections were then incubated in 3% hydrogen peroxide in absolute methanol at room temperature for 10 min, to block endogenous peroxidase activity. After three rinses (each for 5 min) in PBS, the sections were incubated with rabbit anti-human RAD51 antibody (1:75, SCBT, Santa Cruz, CA), diluted in PBS overnight at 4°C, with 150 µl polymerized HRP-anti mouse/rabbit IgG for 30 min at room temperature. The reaction products were visualized with diaminobenzidine (DAB Kit; Zhongshan Goldenbridge Biotechnology), and slides were counterstained with hematoxylin, dehydrated, and evaluated under light microscope. Negative controls were done by omitting primary antibodies. The intensity of staining within the nucleus and the cytoplasm of the cells was assessed. A sample was classified as positive if more than 10% of the cells (malignant or benign) were stained with the RAD51 antibody with intensity greater than 0. A consensus score of expression intensity (1, low; 2, moderate; and 3, strong) was reached by two investigators (Q. Liu, C. Hao) for each sample.

### Statistical Analysis

For each measurement, three or four independent experiments were performed. Results were expressed as mean ± SEM. Statistical calculations were performed using the SigmaPlot 2000 software (Systat Software, San Jose, CA). Differences in measured variables between experimental and control groups were assessed using t-test. *P*<0.05 was considered statistically significant.
